# Expression of p-STAT3 and c-Myc correlates with P2-HNF4α expression in nonalcoholic fatty liver disease (NAFLD)

**DOI:** 10.18632/oncotarget.28324

**Published:** 2022-12-06

**Authors:** Mamoun Younes, Lin Zhang, Baharan Fekry, Kristin Eckel-Mahan

**Affiliations:** ^1^Department of Pathology, George Washington University School of Medicine and Health Sciences, Washington, DC 20037, USA; ^2^Departments of Pathology and Laboratory Medicine, McGovern Medical School at the University of Texas Health Science Center (UTHealth), Houston, TX 77225, USA; ^3^Institute of Molecular Medicine, McGovern Medical School at the University of Texas Health Science Center (UTHealth), Houston, TX 77225, USA

**Keywords:** hepatocyte nuclear factor four alpha, steatohepatitis, immunohistochemistry, hepatocellular carcinoma, isoform

## Abstract

We studied the expression of two hepatocyte nuclear factor 4 alpha (HNF4α) isoforms, p-STAT3. and c-Myc in 49 consecutive liver biopsies with nonalcoholic fatty liver disease (NAFLD) using immunohistochemistry. All 49 biopsies (100%) were positive for nuclear expression of P1-HNF4α. Twenty-eight (57%) cases were positive for P2-HNF4α, 6 (12%) were positive for p-STAT3 and 5 (10%) were positive for c-Myc. All 6 (100%) p-STAT3-positive cases were also positive for P2-HNF4α (*p* = 0.03). p-STAT3-positive cases were more likely to be positive for c-Myc (67% vs. 2%, *p* = 0.0003). Four cases were positive for P2-HNF4α, p-STAT3 and c-Myc. p-STAT3 expression was associated with hypertension (*p* = 0.037). All c-Myc positive biopsies were from patients with obesity, diabetes and hypertension. Only c-Myc expression was associated with advanced fibrosis; three (60%) of the c-Myc positive cases were associated with advanced fibrosis in contrast to 7 (10%) of the 44 c-Myc negative cases (*p* = 0.011). Based on these results, we hypothesize with the following sequence of events with progression of NAFLD: P2-HNF4α expression is followed by expression of p-STAT3 which in turn is followed by the expression of c-Myc. Additional larger studies are needed to confirm these findings.

## INTRODUCTION

Nonalcoholic fatty liver disease (NAFLD) is associated with the metabolic syndrome and is rapidly becoming one of the major causes of hepatic cirrhosis and hepatocellular carcinoma (HCC), although some cases of HCC have developed in non-cirrhotic livers [1–8]. Although the percentage of patients with NAFLD who ultimately progress to fibrosis and later to HCC is relatively small, the number is significant because of the sheer number of patients who have NAFLD. Because there are no reliable biomarkers to predict the risk of HCC in patients with NAFLD, designing effective and cost-effective surveillance programs aimed at prevention and early detection of HCC is difficult, if not impossible. Therefore, there is an urgent need to identify such biomarkers and especially those that may appear at different stages of progression toward HCC.

The protein hepatocyte nuclear factor 4 alpha (HNF4α) has been identified as a central gene in the pathogenesis of nonalcoholic steatohepatitis (NASH), a subset of NAFLD characterized by inflammation with increased risk for cirrhosis and HCC over NAFLD [9]. There are two main isoforms of HNF4α, driven by different promoters, the so-called “P1” and “P2” promoters. P2-HNF4α is expressed predominantly in fetal liver, while the P1-driven isoforms are mainly expressed in the normal adult hepatocytes. Previously, we have shown that P2-HNF4α is induced in the mouse model of diabetic dyslipidemia (the db/db mouse) and in mice with diet-induced obesity. Induction of P2-HNF4α is coincident with altered subcellular localization of P1-HNF4α from the nucleus to the cytoplasm [10]. Loss of P1-HNF4α is associated with an increase in tumorigenesis; with male and female HNF4α-deficient mice acquiring early onset HCC when fed a high fat diet [11]. The same animal model also shows accelerated HCC under conditions of diethylnitrosamine (DEN) induced HCC [12, 13]. Additionally, while P2-HNF4α is not highly expressed in livers of normal adults [14], it has been reported to be expressed in hepatocellular carcinomas [10]. Phosphorylation of STAT3 (p-STAT3) and induction of c-Myc have been proposed to be major contributors to development of HCC [15–19]. The aim of this study was to determine the relationships between p-STAT3, c-Myc and P2-HNF4α expression in biopsies from livers with NAFLD as potential biomarkers of HCC risk.

## RESULTS

**Figure 1:**
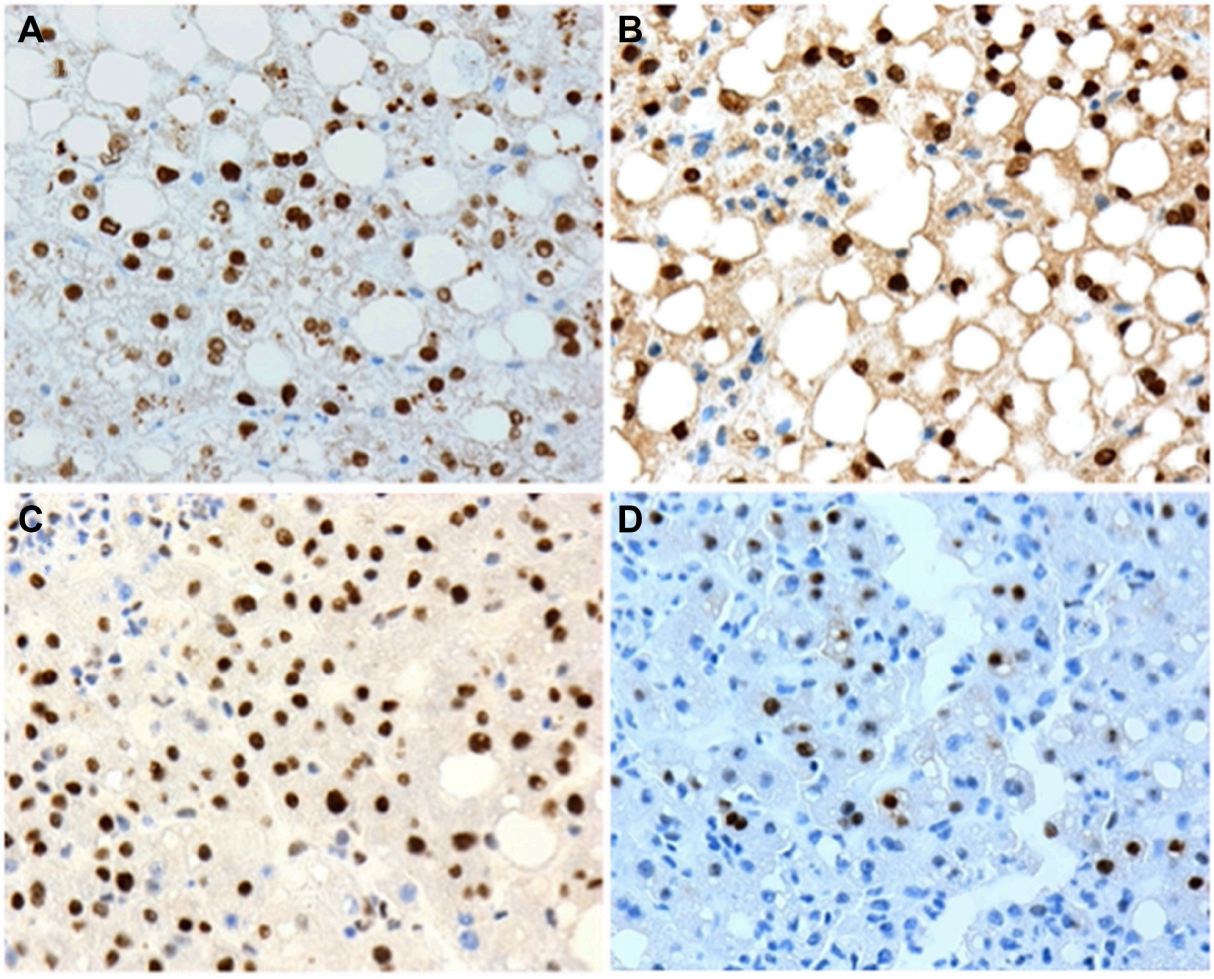
Examples of positive nuclear expression of (**A**) P1-HNF4α; (**B**) P2-HNF4α; (**C**) p-STAT3 and (**D**) c-Myc and human liver biopsies with non-alcoholic fatty liver disease (NAFLD). 20X microscope objective.

The demographic and relevant clinicopathologic characteristics of the 49 patients in this study are shown in Table 1. Examples of positive immunohistochemical staining for the 4 biomarkers used in this study are shown in Figure 1.

**Table 1:** Patient demographics and clinicopathologic information

**Age (years)**	**22–76 (mean 46, median 47)**
Sex	
Males	16
Females	33
Race	
Asian	2
Black	6
Hispanic	6
White	28
Other/unknown	7
Obesity	
Obese	39
Not obese	10
Diabetes	
Diabetic	25
Not diabetic	20
Unknown	4
Blood pressure	
High	30
Normal	19
Steatohepatitis grade	(0–8)
2	12
3	15
4	10
5	10
6	1
7	1
Steatohepatitis stage	(0–4)
0	7
1	17
2	15
3	8
4	2

**Figure 2:**
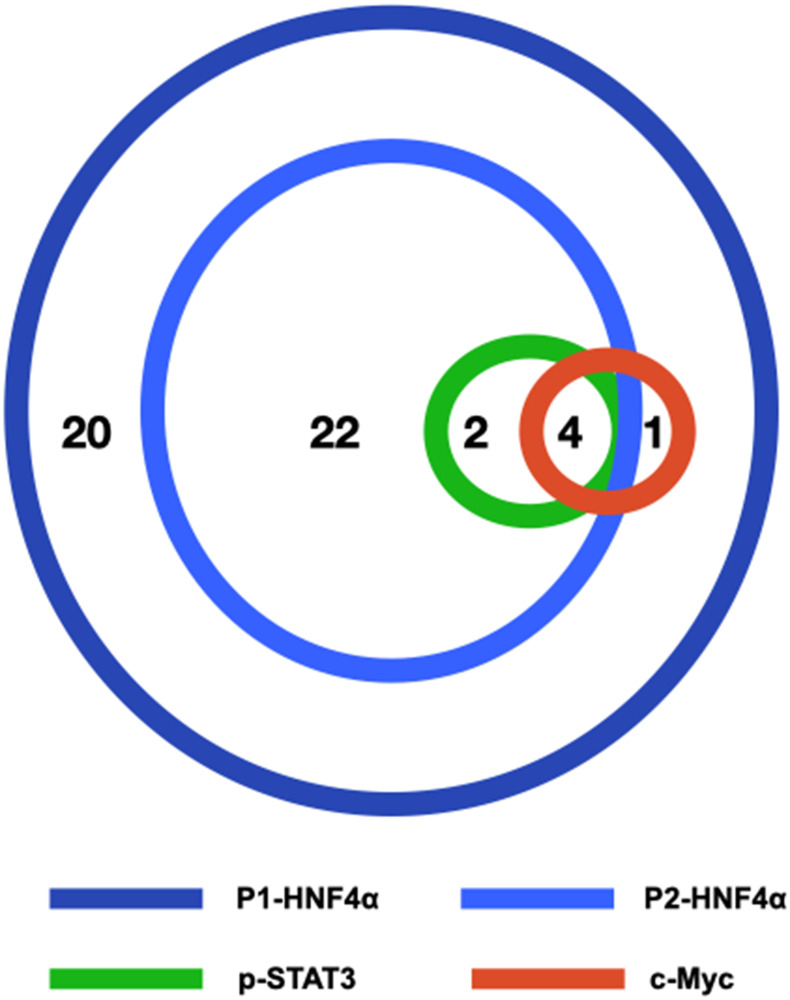
Schematic figure illustrating the relationships of P1-HNF4α, P2-HNF4α, p-STAT3 and c-Myc in our series of 49 liver biopsies with NAFLD. The number of cases positive for one or more biomarkers is shown.

All 49 biopsies were positive for nuclear expression of P1-HNF4α. Twenty-eight (57%) cases were positive for nuclear expression of P2-HNF4α, 6 (12%) were positive for nuclear p-STAT3 and 5 (10%) were positive for nuclear c-Myc. Four of the 49 cases (8%) were positive for the three biomarkers P2-HNF4α, p-STAT3 and c-Myc (Figure 2).

All (100%) p-STAT3 cases were also positive for P2-HNF4α (*p* = 0.024). Four of the 5 (80%) c-Myc positive cases were also positive in p-STAT3 (*p* = 0.001). Four c-Myc positive cases were positive for both P2-HNF4α and p-STAT3. There was no significant correlation between P2-HNF4α, p-STAT3 or c-Myc expression and patient gender or age. The relationships between the expression of P2-HNF4α, p-STAT3 and c-Myc in liver biopsies and components of the metabolic syndrome (obesity, hypertension and diabetes) and liver fibrosis is shown in Table 2.

**Table 2:** Relationships of P2-HNF4α, p-STAT3 and c-Myc expression in liver biopsies and components of the metabolic syndrome and liver fibrosis

	**P2-HNF4α**			**p-STAT3**			**c-Myc**		
**Groups**	**+**	**−**	***p***	**+**	**−**	***p***	**+**	**−**	***p***
Obese	24	15	NS	5	34	NS	5	34	NS
Not obese	4	6		1	9		0	10	
Hypertensive	17	13	NS	6	24	0.037	5	25	0.06
Normotensive	11	8		0	19		0	19	
Diabetic	12	13	NS	3	22	NS	3	22	NS
Not diabetic^*^	15	5		2	18		0	22	
FS^**^ 3–4	4	5	NS	2	7	NS	3	6	0.011
FS 0–2	24	16		4	36		2	38	

^*^Diabetes status was unknown on four patients. Abbreviation: ^**^FS: Fibrosis stage.

All four biopsies that were positive for P2-HNF4α, p-STAT3 and c-Myc at the same time were associated with obesity, diabetes and hypertension and 50% of these were associated with advanced fibrosis however this was not statistically significant likely due to the small number of cases positive for all three markers.

## DISCUSSION

The overall findings from our study suggests that during progression from NAFLD to fibrosis nuclear expression of P2-HNF4α, which in our series is seen in over half of the cases, may be the first event to occur. P2-HNF4α appears to create a permissive environment for phosphorylation of STAT-3 (p-STAT3) in a smaller subset of livers which appears to occur only in biopsies with nuclear expression of P2-HNF4α. We have previously observed that induction of P2-HNF4α is concomitant with a partial shift in nuclear P1-HNF4α, allowing derepression of interleukin 6 (*Il6*) expression, which leads to STAT3 phosphorylation [10]. Though our prior pre-clinical studies suggest an association of P2-HNF4α and obesity and diabetes, in this series there was no significant association between any of the three components of the metabolic syndrome that we have data on in this study (obesity, diabetes and hypertension). There was also no significant association between p-STAT3 and hypertension. Further studies will be needed to determine the extent to which human metabolic disease correlates with P2-HNF4α induction. Neither P2-HNF4α nor p-STAT3 were associated with advanced fibrosis, which is associated with increased risk for HCC compared to the population with NAFLD at large. c-Myc expression appears to occur later than P2-HNF4α and p-STAT3 because: 1, it is only seen in a subset of the patients positive for p-STAT3 (except one patient); 2, it is significantly associated with advanced fibrosis which is known to occur at the end stage in NAFLD progression; and 3, because cases that are positive for all three markers are more likely to be associated with advanced fibrosis. Also of interest, patients with liver biopsies positive for c-Myc or positive for all three markers were all obese, diabetic and hypertensive.

We realize that the cases positive for p-STAT3 and c-Myc are few, so our results should be considered preliminary until validated by larger studies that include follow up biopsies and data on progression to both cirrhosis and HCC. However, if proven accurate, this could be the basis for a multi-biomarker stepwise progression model from NAFLD to HCC which allows tailoring surveillance and follow up programs, specific chemo/dietary preventive trials, and early detection (like increasing frequency of scans or blood tests) to detect HCC at an early and curable stage. Phosphorylation of STAT3 Y705 residue, for example, can be induced by interleukin 6 (IL-6) [20]. Production of IL-6 may be significantly reduced using natural dietary products such as lactoferrin [21] and other recently developed inhibitors of the IL-6/STAT3 signaling pathway [22].

In conclusion, our results suggest that STAT3 phosphorylation occurs in livers with NAFLD only in the subset with P2-HNF4α expression, and c-Myc expression is strongly correlated with STAT3 phosphorylation. We hypothesize that P2-HNF4α expression is an early event in the development of HCC in NAFLD that is followed at a later step by STAT3 phosphorylation and c-Myc expression.

## MATERIALS AND METHODS

The pathology files were searched for all liver biopsies with a diagnosis of “steatosis” or “steatohepatitis” performed over one year period (2016) and the slides and corresponding reports were retrieved and reviewed. After excluding biopsies with any type of tumor (benign or malignant), alcoholic hepatitis, granulomas, viral hepatitis, drug-induced liver injury, cholestatic hepatitis, primary biliary cholangitis, sclerosing cholangitis the remaining biopsies from 49 patients were entered in this study. Information regarding the patient age, gender/sex, obesity, blood pressure, and diabetes were obtained from review of the medical records. The inflammation grade (0–8) and fibrosis stage (0–4) were scored on all biopsies using the Clinical Research Network Histologic Scoring System. Advanced fibrotic stage is considered when the fibrosis stage was 3 or 4.

The anti-human monoclonal P1-HNF4α and P2-HNF4α antibodies were obtained from R&D Systems. The anti-STAT3 (phospho Y705) and anti-c-Myc antibodies were obtained from Abcam. Sections were stained for P1-HNF4α, P2-HNF4α, p-STAT3 and c-Myc using standard immunohistochemistry staining (IHC) protocols on a Dako autostainer (Agilent, Santa Clara, CA, USA). Sections of formalin-fixed and paraffin-embedded cell lines that are known to be positive or negative for each of the biomarkers tested were included in every batch of staining as positive and negative controls, respectively. Staining was considered positive if the intensity was medium or strong and in more than 1% of the cells (scattered rare positive cells considered negative).

The staining results were correlated with each other and with clinicopathologic features. Statistical analysis was performed using Wizard 2 statistical software for the Mac (Evan Miller, Apple App Store) using the Chi-square test (*t*-test used for exploring correlations with patient age); *p* < 0.05 is considered significant.

The study was approved by the Institutional Review Board of the University of Texas Health Science Center at Houston.

## Abbreviations

FS: fibrosis stage
HNF: hepatocyte nuclear factor
IHC: immunohistochemistry
NAFLD: non-alcoholic fatty liver disease
NASH: non-alcoholic steatohepatitis
NS: not significant
p-STAT3: phospho-STAT3
